# The Emptiness Within: A Case of Empty Sella Syndrome

**DOI:** 10.7759/cureus.28941

**Published:** 2022-09-08

**Authors:** Afees Ahamed M A, Sahana Shetty, Sakinya Hegde, Pratibha Prasannan

**Affiliations:** 1 General Medicine, Kasturba Medical College, Manipal, IND; 2 Endocrinology, Kasturba Medical College, Manipal, IND

**Keywords:** central hypocortisolism, hyponatremia, central hypothyroidism, panhypopituitarism, empty sella syndrome

## Abstract

Empty Sella syndrome (ESS) is characterized by the sella turcica being filled with cerebrospinal fluid (CSF), leading to partial or total compression of the pituitary gland, often resulting in hormonal deficiencies. It can be primary or secondary. In patients presenting with complaints of generalized weakness and fatiguability, with multiple episodes of prior hospitalizations, a thorough history and evaluation can lead to a diagnosis. We report a case of a 50-year-old lady with recurrent admissions for hyponatremia. Based on biochemical parameters and brain imaging, she was diagnosed to have ESS. We report this case to highlight the various diagnostic challenges associated with panhypopituitarism and the importance of having a high clinical suspicion, as the treatment is simple and lifesaving.

## Introduction

Empty Sella syndrome (ESS), also known as arachnoidocele, is a disorder in which the subarachnoid space herniates into the sella turcica causing compression and flattening of the pituitary gland. It is a rare syndrome with incidence ranging from 5.5% to 12% of autopsy cases, up to 12% in patients undergoing neuroimaging [1]. It is more common in females with a sex ratio of 4-5:1 [1]. Empty sella is present in approximately 70% of patients with idiopathic intracranial hypertension (IIH) [2]. Compression of pituitary parenchyma, along with involvement of pituitary stalk can cause hormonal deficiencies of varying degrees. These can lead to a variety of clinical presentations, often making the family to consider these symptoms as being psychogenic, leading to delay in appropriate consults/referrals. We report a case of a middle-aged lady, who had history of multiple outpatient consults and inpatient admissions, being diagnosed as a case of ESS upon proper evaluation. Diagnosis is pivotal as treatment is often simple and very effective.

## Case presentation

A 50-year-old lady presented to the emergency department with complaints of loose stools and vomiting for three days. There was history of cessation of menses at the age of 35 years along with multiple prior outpatient consultations for generalized weakness, fatiguability, and recurrent admissions for hyponatremia associated with acute illness. Her repeated hospitalizations had made the family to consider her as being psychogenic. On examination, she was drowsy with pale, dry skin, facial puffiness, loss of lateral eyebrows and non-pitting pedal edema, with a heart rate of 108/min and a blood pressure (BP) recording of 70/50 mmHg. Laboratory investigations done are shown in Table 1.

**Table 1:** Laboratory investigations. CBC, complete blood count; Hb, hemoglobin; Hct, hematocrit; WBC, white blood cell; TSH, thyroid stimulating hormone; ACTH, adrenocorticotropic hormone; FSH, follicle stimulating hormone; RBS, random blood sugar; HBA1C, hemoglobin A1C

Investigations	Values	Reference range
CBC		
Hb (g/dL)	9.6	12-15
Hct (%)	28	34-46
WBC count (/µL)	15.4 x 10^3^	(4-10) × 10^3^
Platelet count (/µL)	175 x 10^3^	(150-400) × 10^3^
Serum laboratory tests		
Na^+ ^(mmol/L)	101	136-144
K^+ ^(mmol/L)	3.9	3.6-5.1
TSH (µIU/mL)	6.9	0.27-4.2
T3 (ng/mL)	0.36	0.8-2
T4 (µg/dL)	2.03	5.1-14.1
FT4 (ng/dL)	0.3	0.93-1.71
Cortisol (µg/dL)	0.93	4.8-19.5 (morning)
ACTH (pg/mL)	<1	7.2-63.3
FSH (mIU/mL)	11.1	25.8-134.8 (post-menopausal)
Others		
Plasma RBS (mg/dL)	98	70-140
HbA1C	5.6	<5.7

Biochemical investigations revealed hyponatraemia, low cortisol levels with low adrenocorticotropic hormone (ACTH) suggestive of central hypocortisolemia, low T4 and FT4 levels with high normal thyroid stimulating hormone (TSH) suggestive of central hypothyroidism, low follicle stimulating hormone (FSH) in the presence of amenorrhea suggestive of central hypogonadism -- pointing to panhypopituitarism. In patients with central hypothyroidism serum free T4 is low but TSH may be low, normal, or slightly elevated up to 10 µIU/mL [3-4].

**Figure 1:**
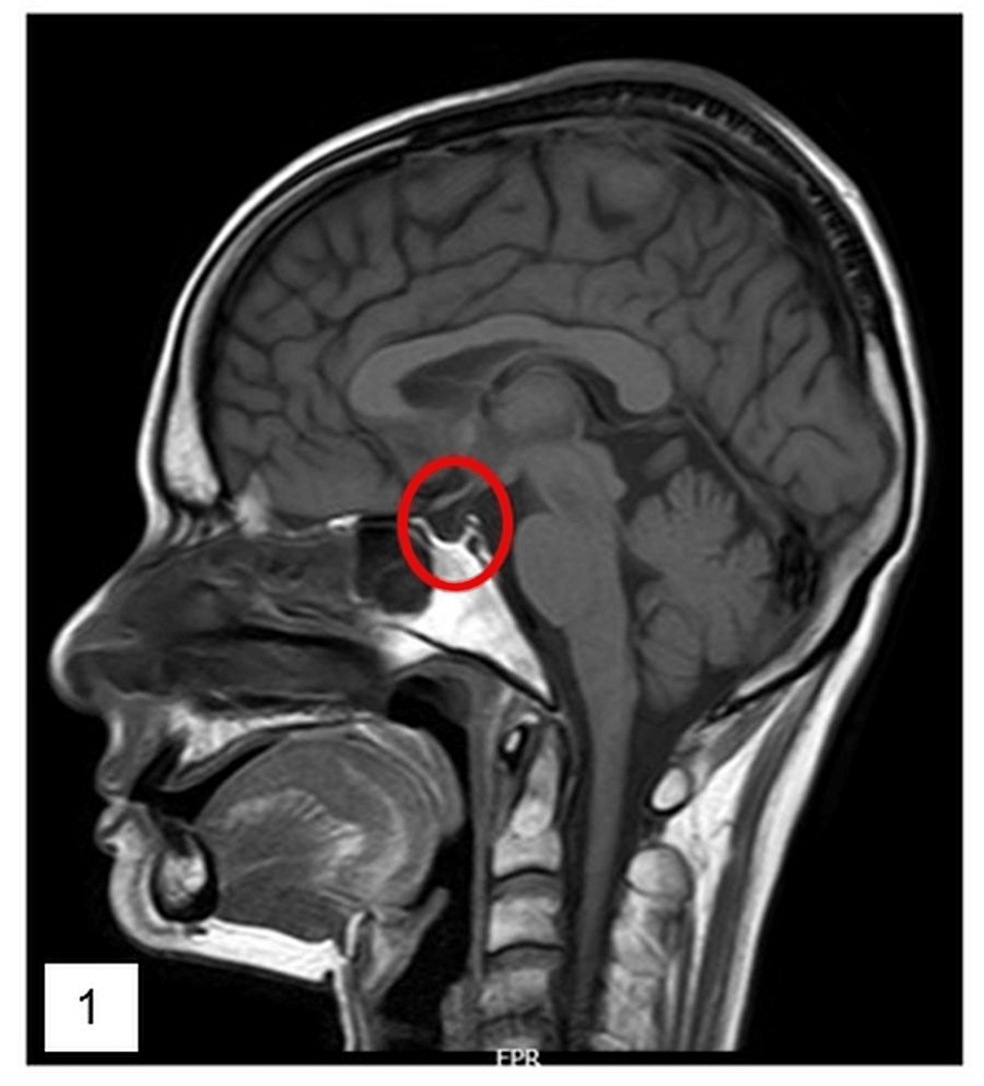
T1 weighted image - sagittal section - shows empty sella filled with CSF, with thin rim of pituitary gland along the wall of the cavity. CSF, cerebrospinal fluid

**Figure 2:**
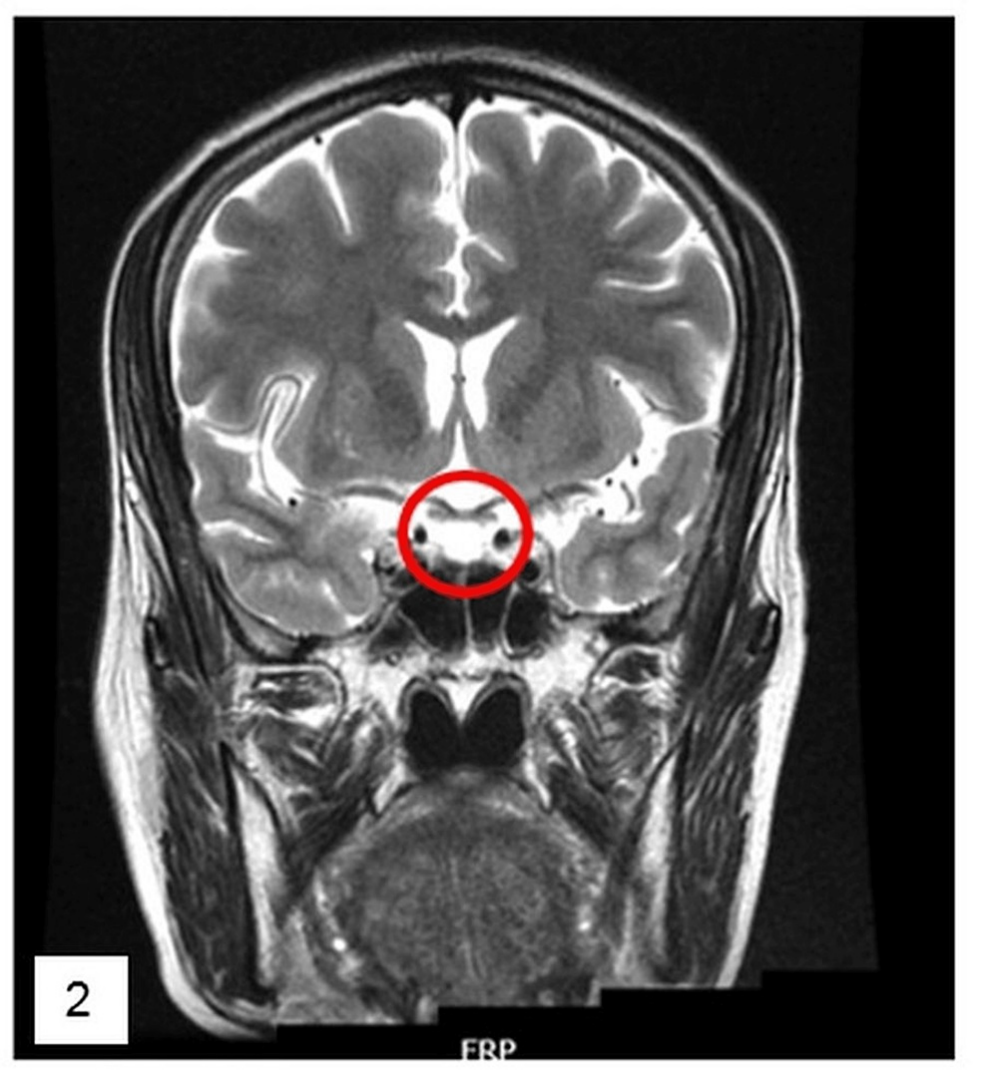
T2 weighted image - coronal view - CSF filling the empty sella. CSF, cerebrospinal fluid

Imaging with MRI pituitary showed thinning of the pituitary gland with cerebrospinal fluid (CSF) filled sella, suggestive of empty sella (Figures 1-2).

Hence a diagnosis of panhypopituitarism secondary to ESS was made. The patient was initially resuscitated with IV fluids, started on parenteral hydrocortisone and oral thyroxine, following which her sensorium improved and she attained hemodynamic stability along with normalization of serum sodium levels. She was later discharged on oral steroid and thyroxine supplementation. The patient had improvement of hypothyroidism features during subsequent visits and is currently doing well.

## Discussion

Empty sella syndrome is defined as intrasellar herniation of the suprasellar subarachnoid space leading to compression of the pituitary gland often due to a defect in diaphragma sellae along with increased CSF pressure [5]. It can be primary or secondary. Primary ESS occurs due to a small anatomical defect in the diaphragma sellae, along with increased CSF pressure causing the gland to flatten out along the interior walls of the sella turcica cavity. It occurs primarily in middle-aged women who are obese and hypertensive [6]. The prevalence of primary empty sella ranges from 2% to 20% [7]. In secondary ESS, the pituitary gland regresses often after an injury, apoplexy, hypophysitis, surgery, or radiation therapy. The estimated prevalence of hypopituitarism in ESS is 52%. The somatotropic and gonadotropic axis are the most affected, followed by the thyrotropic and corticotropic axis [7]. 

Empty sella can be an incidental radiological finding and may usually not present with symptoms [8]. However, when it is associated with endocrine, neurologic, ophthalmologic, or psychiatric symptoms, a diagnosis of ESS is made [9]. Overweight and obesity are observed in majority of the patients, particularly in females, in fact, around the 50% of females with primary EES are obese [10]. Menstrual abnormalities are noted in 40% of females, along with galactorrhoea in 26% and hypertrichosis in 18% [10]. Around 12% of male patients, present with gynecomastia and close to 53% have sexual disturbances [10]. The presence of raised intracranial pressure (ICP) predominantly leads to neurological and ophthalmological signs in ESS [1]. 

Empty sella can be confirmed through magnetic resonance (MR) study of sellar and suprasellar regions and with CT in whom MRI is contraindicated. Imaging reveals enlarged bony sella, with residual pituitary gland of semi-lunate morphology, flattened against the sellar floor, along with intrasellar CSF filling. Basal pituitary hormone measurements must be done for all cases and dynamic tests, if indicated [1]. Ophthalmological examination gives an indirect assessment of the optic nerve morphology and thus about ICP. Treatment involves replacement of deficient hormones in appropriate temporal sequence. It is recommended that hormonal replacement therapy starts with hydrocortisone, followed by thyroid hormone supplementation. Once the patient’s condition stabilizes, replacement of sex hormones can be done if indicated. With appropriate therapy, patients have resolution of symptoms and quality of life improves drastically.

## Conclusions

The case reinforces the need for appropriate investigations and interpretations. Hypocortisolemia must be considered and evaluated in every patient presenting with hyponatraemia. In presence of central hypocortisolaemia, evaluation of other pituitary axis and pituitary imaging must be done to ascertain the underlying etiology -- such as ESS. Further, if only serum TSH is measured, central hypothyroidism may be missed, as it may be normal, low, or high normal in central hypothyroidism. Hence free T4/total T4 must also be measured. Measuring serum FSH in patients with early menopause may help in diagnosing rare cases of central hypogonadism if FSH is below the menopausal range. Keeping a high clinical suspicion and diagnosing panhypopituitarism is vital as the treatment is simple and lifesaving. Although the diagnosis of hypopituitarism is challenging, the treatment is straightforward and involves replacement with glucocorticoids, thyroxine, and sex steroids which is lifesaving in these patients.
